# Differences in Cadmium Accumulation, Detoxification and Antioxidant Defenses between Contrasting Maize Cultivars Implicate a Role of Superoxide Dismutase in Cd Tolerance

**DOI:** 10.3390/antiox10111812

**Published:** 2021-11-15

**Authors:** Aya Mahmoud, Hamada AbdElgawad, Badreldin A. Hamed, Gerrit T.S. Beemster, Nadia M. El-Shafey

**Affiliations:** 1 Department of Botany and Microbiology, Faculty of Science, Beni-Suef University, Beni-Suef 62511, Egypt; ayayehia@science.bsu.edu.eg (A.M.); hamada.abdelgawad@science.bsu.edu.eg (H.A.); badr.hamed@science.bsu.edu.eg (B.A.H.); 2 Integrated Molecular Plant Physiology Research (IMPRES), Department of Biology, University of Antwerp, 2020 Antwerp, Belgium; gerrit.beemster@uantwerpen.be

**Keywords:** Cd, maize, detoxification, oxidative stress, superoxide dismutase, Cd-tolerance mechanisms

## Abstract

Cadmium (Cd), a readily absorbed and translocated toxic heavy metal, inhibits plant growth, interrupts metabolic homeostasis and induces oxidative damage. Responses towards Cd-stress differ among plant cultivars, and the complex integrated relationships between Cd accumulation, detoxification mechanisms and antioxidant defenses still need to be unraveled. To this end, 12 Egyptian maize cultivars were grown under Cd-stress to test their Cd-stress tolerance. Out of these cultivars, tolerant (TWC360 and TWC321), moderately sensitive (TWC324) and sensitive (SC128) cultivars were selected, and we determined their response to Cd in terms of biomass, Cd accumulation and antioxidant defense system. The reduction in biomass was highly obvious in sensitive cultivars, while TWC360 and TWC321 showed high Cd-tolerance. The cultivar TWC321 showed lower Cd uptake concurrently with an enhanced antioxidant defense system. Interestingly, TWC360 accumulated more Cd in the shoot, accompanied with increased Cd detoxification and sequestration. A principal component analysis revealed a clear separation between the sensitive and tolerant cultivars with significance of the antioxidant defenses, including superoxide dismutase (SOD). To confirm the involvement of SOD in Cd-tolerance, we studied the effect of Cd-stress on a transgenic maize line (TG) constitutively overexpressing *AtFeSOD* gene in comparison to its wild type (WT). Compared to their WT, the TG plants showed less Cd accumulation and improved growth, physiology, antioxidant and detoxification systems. These results demonstrate the role of SOD in determining Cd-tolerance.

## 1. Introduction

Cadmium (Cd) is recognized as an important pollutant due to its high toxicity, even at low concentrations, and high solubility in water [1,2]. It is considered as one of the most readily absorbed and most rapidly translocated heavy metals [3,4]. Cd accumulation in plants inhibits cell division in meristems [5,6]. Cd also interrupts photosynthesis and other metabolic reactions causing the generation of reactive oxygen species (ROS), resulting in oxidative stress [7,8].

To avoid injuries induced by Cd toxicity, plants have developed several defense mechanisms [1]. These mechanisms include inhibition of Cd influx, stimulation of efflux, compartmentation, sequestration and detoxification [6]. In this regard, chelators, reduced glutathione (GSH) and phytochelatins (PCs) are utilized in chelation and sequestration of Cd in vacuole. In addition to PCs, which are glutathione oligomers that bind metals and sequester them to the vacuole, glutathione-S-transferase (GST) is also involved in metal detoxification by catalyzing glutathione-metal conjugation [9,10].

Cd is a redox inactive heavy metal that induces ROS, including superoxide radical (O_2_^•−^), hydrogen peroxide (H_2_O_2_) and hydroxyl radical (^•^OH) [11]. The superoxide radical (O_2_^•−^) is mainly produced in chloroplast by the leakage of electrons to molecular oxygen on the electron acceptor side of PSII or the incomplete oxidation of water on the electron donor side of PSII [12]. Cd was reported to inhibit both the donor and acceptor sides of PSII, the former by replacing Ca on oxygen-evolving complex (OEC) and the latter by reducing the rate of electron transport from the primary quinone acceptor (QA) to secondary quinone acceptor (QB) [13,14,15]. Although causing non-extensive damage, the superoxide radical as a precursor of most of ROS, when generated, may initiate the formation of more toxic and reactive radicals (singlet oxygen; ^1^O_2_ and ^•^OH), causing lipid peroxidation and protein disfunction. The enzyme superoxide dismutase (SOD) is responsible for dismutating O_2_^•^^−^ into O_2_ and H_2_O_2_. Thus, SOD is a key control in the ROS scavenging and the antioxidant defense. Two of SOD isozymes are localized in chloroplast; Cu/Zn-SOD is attached to the thylakoid membrane at the vicinity of PSI and FeSOD is attached to the stromal side of the thylakoid membrane close to PSII [16]. The expression of FeSOD is upregulated in response to Cd in perennial ryegrass (*Lolium perenne* L.) and *Arabidopsis thaliana* [17,18,19], suggesting a key role in the defense to this stress. Consistently, transgenic tobacco plants with decreased chloroplastic FeSOD exhibited increased O2^•−^ production and D1 degradation [20]. Overall, increased antioxidant defense is an adaptive response of plants to mitigate Cd-stress [21,22]. These increases support plants to sustain their cellular redox status and mitigate the damage caused by the accumulated toxic metal and oxidative stress [23].

Maize (*Zea mays* L.) is one of the most abundant cereal crops [24], suitable for tropical to temperate climates. Being a rich source of nutrition (72% starch, 10% protein, 8.5% fiber and 4.8% edible oil), maize is a major source of food, sugar, cooking oil and animal feed [25]. Phytoremediation and utilization as bioenergy crop are additional benefits of the maize plant [26,27,28]. The responses of maize to Cd have been studied extensively, starting off with growth in relation with Cd uptake and accumulation, moving to detoxification and sequestration of Cd and ending with the antioxidative system and its involvement in Cd tolerance, in addition to the gene expression studies [27,29,30,31,32,33,34]. However, the complex integrated relations among antioxidant defenses require Cd accumulation and detoxification mechanisms to still be unraveled.

In this study, we screened 12 cultivars to evaluate their tolerance in terms of fresh and dry biomass. We selected four cultivars differing in their tolerance and studied their Cd uptake, translocation and accumulation in both shoot and root. We also studied their antioxidant mechanisms in a trial to find if there is a relation among Cd translocation and accumulation in shoot, glutathione (GSH) as a thiol compound involved in Cd detoxification and the antioxidant defense system. Our results implicated a role for the SOD enzyme in plant resistance to Cd stress. To validate its role, maize transgenic line overexpressing the *FeSOD* gene from *Arabidopsis* was tested for its tolerance against Cd stress. Our results indicated that overexpression of SOD improved plant growth, physiology and biochemistry under Cd stress.

## 2. Materials and Methods

### 2.1. Plant Material and Stress Treatment

In this experiment, 12 maize cultivars, as commonly cultivated in Egypt, were tested in pot experiment to select those are the most tolerant and the most sensitive cultivars to Cd-stress. Seeds of the investigated cultivars were obtained from the Agriculture Research Center, Giza, Egypt. The experiment was carried out during May–June 2018 in Beni-Suef University, in an open field condition and under natural light in a randomized complete block design. The temperature ranged between 36 °C as maximum and 27 °C as minimum. Plastic pots (14 cm diameter and 15 cm height) were filled with one kg sand: clay soil (3:1) for each pot. Cadmium was applied as different concentrations (0.0, 80 and 160 mg of Cd Kg^−1^ soil, using CdCl_2_·2.5H_2_O), representing control, mild and severe Cd-stress, respectively. Five pots were prepared per each treatment and Cd was incorporated into the soil before sowing. Ten seeds were sown per pot and pots were irrigated regularly with tap water. Seedlings were thinned after immergence to keep four seedlings per pot. After 50 days (of sowing), maize growth was estimated as length and fresh and dry weights of shoot and root. Tolerance index (TI) was estimated for each parameter (length, fresh weight and dry weight) in both shoot and root of the investigated cultivars. For example, to calculate TI for dry weight: as TI = [(DWM + DWS)/DWC]/2: where DWM: dry weight at mild stress, DWS: dry weight at severe stress and DWC: dry weight of control. Cultivars scored the highest and lowest shoot and root TI were selected as tolerant and sensitive to be evaluated for their growth and physiological response under cadmium stress. Four cultivars (sensitive; SC128 and TWC324, and tolerant; TWC321 and TWC360) were selected according to their lowest and highest TI, respectively. The selected cultivars were subjected to Cd-stress in an experiment that was carried out during May–June 2019 in the same conditions as mentioned above with temperature range of 35 °C for maximum and 22 °C for minimum. Fifty days after sowing, morphological criteria were estimated. Shoot and root samples were dried in an oven at 70 °C and kept for estimation of Cd content. Fresh ones were used in the rest of the biochemical analyses.

To test the effect of enhancing the antioxidants on Cd-tolerance, in association with Cd accumulation, we used a *FeSOD* overexpressing maize transgenic line and its wild type (H99). The transgenic line was induced by overexpressing the *FeSOD* gene from Arabidopsis *(Arabidopsis thaliana)* under the control of the cauliflower mosaic virus 35S promoter, the backcross used were of Pa91 × H99 to the H99 parent. Seeds of wild and transgenic lines were provided by Frank Van Breusegem [35]. Seeds were planted on peat potting medium (57% soil water content, Jiffy Products International B.V., the Netherlands) after applying Cd-stress (control, mild and severe) using cadmium sulphate (CdSO_4_·8/3H_2_O; 0.0, 46.5 and 372.1 mg Cd Kg^−1^ dry soil). Pots were covered with plastic wrap, irrigated daily with tap water and maintained at the original soil water content. Seedlings were grown for 24 days after sowing in the growth chamber under controlled conditions (16-h day/8-h night, 25 °C/18 °C day/night, 300–400 μEm^−2^s^−1^ photosynthetically active radiation and provided by high-pressure sodium lamps). Growth parameters (fresh and dry weights) were estimated and fresh shoots were kept at −80 °C for biochemical analyses.

### 2.2. Cadmium Content, Bioconcentration Factor (BCF) and Translocation Factor (TF)

Cadmium content in both shoot and root was estimated using the protocol of Cottenie, et al. [36], while that of Soltanpour [37] was followed to estimate the available Cd in soil. Both were methods based on using inductively coupled plasma (ICP) spectrometer. Bioconcentration factor (BCF) and translocation factor (TF) were estimated [38] as the following:

Bioconcentration factor (BCF) = Cd_plant_/Cd_soil_, where Cd_plant_ is Cd concentration in harvested plant material (mg kg^−1^) and Cd_soil_ is Cd concentration in soil (mg kg^−1^).

Translocation factor (TF) = Cd_shoot_/Cd_root_, where Cd_shoot_ is Cd concentration in plant shoot (mg kg^−1^) and Cd_root_ is Cd concentration in root (mg kg^−1^).

### 2.3. Detoxification

Glutathione-S-transferase (GST) activity was assayed as described in Habig, et al. [39]. The activity was assayed following the change of absorbance at 340 nm due to conjugation of reduced glutathione (GSH) with 1-chloro-2,4-dinitrobenzine (CDNB). The enzyme activity was expressed as μmol CDNB mg^−1^ protein min^−1^. Phytochelatins content was measured according to de Knecht, et al. [40] by extraction of total non-protein thiols of plant samples in a mixture of (5% sulfosalicylic acid and Ellman’s reagent) and measured using spectrophotometer at 412 nm. Phytochelatins content was expressed as the difference between total non-protein thiols and total glutathione content that was estimated as described below, and expressed as μmol g^−1^ FW.

### 2.4. Photosynthesis

The light saturated photosynthetic rate was determined with a portable photosynthesis system (LI-6400; LI-COR). The temperature and CO_2_ concentration in the leaf chamber were kept at 25 ± 0.5 °C and 400 μmol mol^−1^, respectively. All parameters were estimated inside the growth room at noon [41]. Concerning stomatal conductance (*gs*), this was determined using a Leaf Porometer (Model SC-1, Decagon Devices, Inc., Hopkins, WA, USA) [34]. Fully expanded dark adapted leaves were used for chlorophyll fluorescence determination with FMS-2 pulse-modulated fluorometer (Hansatech Instruments, Norfolk, UK). The minimal and maximal fluorescence (F_0_, F_m_) were assayed for 30 min and photochemical efficiency of PSII was calculated as F_v_/F_m_, where F_m_ (maximal variable fluorescence) = F_m_ − F_0_. Photosynthetic pigments were extracted and determined according to Markwell, et al. [42]. The contents of total chlorophyll (Chl a + b) and carotenoids were calculated [43] and expressed as μg pigment g^−1^ FW.

### 2.5. Oxidative Stress

To reveal the oxidative stress induced by the uptake and accumulation of Cd, the content of hydrogen peroxide (H_2_O_2_) as well as lipid peroxidation and protein oxidation were assayed. Hydrogen peroxide (H_2_O_2_) was extracted in fresh tissue using 0.1% TCA and estimated by the reaction with 1M potassium iodide (KI). The absorbance was read at 390 nm [44], and concentration was expressed as μmol g^−1^ FW. Malondialdhyde (MDA), a product of lipid peroxidation and a thiobarbituric acid reactive substance (TBARS) was estimated according to Jambunathan [45]. Fresh samples were homogenized with trichloroacetic acid (TCA) and centrifuged to extract MDA. The supernatant was assayed with TBA. The content of MDA was expressed as μmol MDA g^−1^ FW. Protein oxidation was measured through carbonyl quantification [46], and the concentration was expressed as nmol mg^−1^ protein.

### 2.6. Enzymatic Antioxidants

By using a pre-cooled mortar and pestle, a fresh maize sample (0.2 g) was pulverized in liquid nitrogen and extracted with 1.2 mL of 50 mM potassium phosphate buffer (pH 7.8) containing 1 mM EDTA.Na_2_. Centrifugation was carried out at 15,000 rpm for 20 min at 4 °C, and the supernatant was used for the assay of total soluble protein and activity of antioxidant enzymes. Total soluble protein was measured using Coomassie blue G250 reagent [47] and bovine serum albumin was used as standard. Super oxide dismutase (SOD) activity was estimated by monitoring the inhibition in the reduction of nitro blue tetrazolium (NBT) into blue color by the accumulated superoxides [48]. The activity was expressed as unit mg^−1^ protein min^−1^, where unit (U) is the amount of enzyme needed to induce 50% inhibition in the rate of NBT photoreduction. The method of Elavarthi and Martin [49] was followed for estimation of catalase (CAT) activity by measuring the decrease in absorbance at 240 nm due to the decomposition of H_2_O_2_. The enzyme activity was expressed in terms of mM H_2_O_2_ mg^−1^ protein min^−1^. The method of Kumar and Khan [50] was used for assaying peroxidase (POX) activity by estimating the oxidation of pyrogallol by H_2_O_2_ into pulpurogallin at 430 nm. The activity of the enzyme was expressed as μ mole pulpurogallin mg^−1^ protein min^−1^.Ascorbate peroxidase (APX), dehydroascorbate reductase (DHAR), monodehydroascorbate reductase (MDHAR), and glutathione reductase (GR) were assayed in a semi- high through put set up [51]. Enzyme activities were measured in extracts provided from 0.1 g of frozen plant material that was homogenized with 1 mL of extraction buffer: 50 mM MES/KOH (pH 6.0) containing 0.04 M KCl, 2 mM CaCl_2_, and 1 mM ascorbic acid (ASC). The activities of APX, MDHAR, DHAR, and GR were assayed in a microplate following the method of Murshed, et al. [52]. The activity of APX activity was assayed by monitoring the decrease absorbance at 290 nm and calculated from the 2.8 mM^21^ cm^21^ extinction coefficient. GR activity was estimated following the change in absorbance at 340 nm and calculated from the 6.22 mM^21^ cm^21^ extinction coefficient. The activity of glutathione peroxidase (GPX) was determined as described by Drotar, et al. [53], in a coupled enzyme analyze with GR, measuring the decrease in NADPH absorbance at 340 nm and calculated from the 6.22 mM^21^ cm^21^ extinction coefficient. Enzymes activities were expressed as μ mol mg^−1^ protein min^−1^.

### 2.7. Non-Enzymatic Antioxidants

Total antioxidant capacity was measured following the Benzie and Strain [54] method for ferric reducing antioxidant power (FRAP) quantification. About 0.2 g frozen samples were ground in liquid nitrogen and then extracted in 2 mL 80% ethanol (ice cold). FRAP assay reagent, containing 0.3 M acetate buffer (pH 3.6), 0.01 mM TPTZ in 0.04 mM HCl and 0.02 M FeCl·3.6H_2_O was mixed in equal volume with the extract. The absorbance was read at 600 nm using a microplate reader (Synergy Mx, Biotek Instruments Inc., Winooski, VT) and FRAP was calculated using trolox standard curve. Flavonoid extraction was performed by homogenizing fifty mg of plant tissues in 0.5 mL ethanol (80% *v*/*v*) and supernatants were collected after centrifugation. Flavonoids concentration was assayed following the modified AlCl_3_ colorimetric method and expressed as mg quercetin g^−1^ FW [55]. About 0.1 g frozen plant tissue was homogenized in a MagNALyser (Roche, Vilvoorde, Belgium), then extracted in ice-cold phosphoric acid (6% *v*/*v*). Then, reduced ascorbate (ASC) and glutathione (GSH) contents were analyzed using HPLC as described in Potters, et al. [56]. HPLC (Shimadzu, ’s-Hertogenbosch, The Netherlands) (reverse phase conditions, Particil Pac 5 μm column material, length 250 mm, i.d. 4.6 mm). An in-line diode array detector (DAD, SPD-M10AVP, Shimadzu, Co., Kyoto, Japan) was used for confirming peaks identity. After reducing the samples using 40 mM DTT, Total contents of ASC and GSH were measured as well as the ASC and GSH redox status, which were expressed as the value of reduced divided by total contents of ASC and GSH, (ASC/TASC) and (GSH/TGSH), respectively, and expressed as μmol g^−1^ FW. Tocopherols were extracted in fresh samples using hexane. After centrifugation, the extract was dried using (CentriVap concentrator, Labconco, Kansas City, MO, USA) and then resuspended in hexane once more. Tocopherol separation and quantifications were performed using HPLC analysis. Dimethyl tocol (DMT) was used as internal standard (5 ppm). Data were analyzed through Shimadzu ClassVP6.14 software, and tocopherols concentration was expressed as mg g^−1^ FW.

### 2.8. Statistical Analysis

Each experiment was repeated twice, and since data were in the same trend, results of the first were shown. Two-way ANOVA was applied to study the effect of the two factors, Cd-stress (Cd) and cultivars (C), and their interaction (Cd × C). For the experiments with the transgenic line, two-way ANOVA was applied for the two factors; Cd-stress (Cd), and SOD and their interaction (SOD × Cd). Significant differences (*p* < 0.05) among means at stress levels within the individual cultivar were compared by Duncan’s multiple range test. Data were statistically analyzed by using SPSS (V16 for windows, Chicago, IL, USA). Multi Experimental Viewer (TM4 software package, http://mev.tm4.org, accessed on 10 October 2021) was applied to generate the principal component analysis (PCA). Differences between WT and TG within each Cd-stress level were analyzed using Student’s *t*-test at *p* < 0.05.

## 3. Results

### 3.1. Screening of Maize Cultivars for Cd-Stress Tolerance

In order to identify potential tolerance mechanisms, we first screened twelve cultivars for their Cd-tolerance. The cultivars were tested in a pot experiment in open field conditions and Cd was applied as different concentrations (0.0, 80 and 160 mg of CdCl_2_·2.5H_2_O Kg^−1^ soil), representing control, mild and severe Cd-stress, respectively. The cultivars were screened according to the morphological parameters (Figures S1–S3) and tolerance index (TI; Table S1). The results indicated that TWC360 had the highest tolerance index followed by TWC321, which exhibited low Cd sensitivity regarding shoot and root biomass. In contrast, SC128 showed a sensitive response by scoring the lowest TI (Table S1), in addition to the strong inhibition in its shoot (−48.9%) and root (−52.2%) dry biomass in relation to control (Figure S3). TWC324 also showed a sensitive behavior but to a lower extent than SC128 in terms of most of the parameters mentioned above.

**Figure 1:**
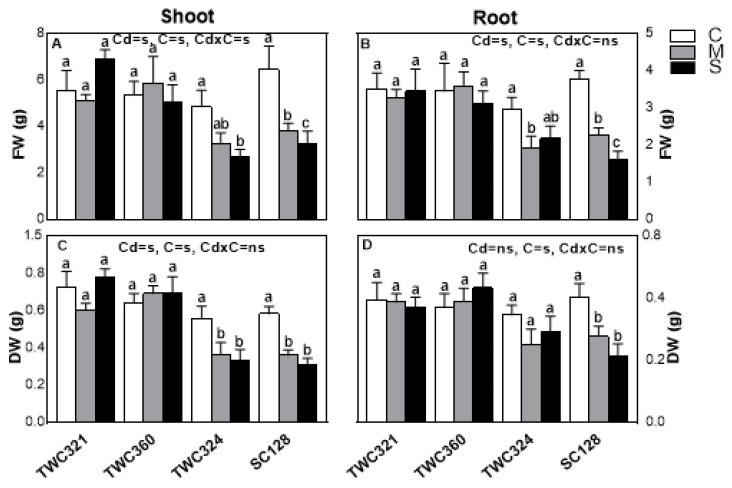
Effect of Cd-stress (C: control, M: mild and S: severe) on (**A**) fresh weight (FW) of shoot, (**B**) fresh weight of root, (**C**) dry weight (DW) of shoot and (**D**) dry weight of root of maize shoot and root respectively. Values are expressed as means ± SE (*n* = 5). Bars with at least one similar letter within each cultivar indicate non-significant difference (*p* ≤ 0.05). Two-way ANOVA was applied to study the effect of the two factors, Cd-stress (Cd) and cultivars (C), as well as their interaction (Cd × C).

After screening, four cultivars differing in their Cd-tolerance were selected. The cultivars (TWC360 and TWC321 as tolerant, TWC324 as moderately sensitive and SC128 as sensitive cultivar) were regrown in the following season (May 2019) to confirm the results of the screening experiment and to study the mechanisms underlying their differing tolerance. The cultivars were investigated for their morphological and physiological responses as well as Cd uptake and accumulation under Cd-stress. The four cultivars showed a differential growth of their shoots and roots in response to Cd-stress (Figure 1A–D) that could be linked to their different ability to utilize Cd-tolerance mechanisms. Consistent with the primary screen, TWC360 showed the most tolerant behavior followed by TWC321, as they recorded a non-significant change in their fresh and dry biomass of shoots and roots (Figure 1A–D). In contrast, the cultivar SC128 was the most sensitive one that recorded 52.1% and 49.2% inhibition in shoot and root dry weights, respectively, under severe Cd-stress (Figure 1C,D). Although the shoot dry weight of TWC324 was declined, its root did not show a significant change, revealing a less sensitive behavior under severe Cd stress.

### 3.2. Differential Cd Accumulation and Detoxification in Tolerant and Sensitive Cultivars

To elucidate whether the variation in tolerance of the investigated cultivars is related to their uptake, translocation and accumulation of cadmium, we estimated cadmium content in shoot and root, in addition to the bioconcentration factor (BCF) and translocation factor (TF). The results showed differences among the four investigated maize cultivars concerning Cd uptake (BCF), Cd translocation from root to shoot (TF) and Cd content in both root and shoot. While SC128 accumulated the highest cadmium content in the shoot, the highest content in the root was found in TWC360 at severe Cd-stress (Table 1). Moreover, TWC360 showed a lower level of Cd translocation, when compared to SC128. Interestingly, both showed similar bioconcentration factors (Table 1). The cultivar TWC321 showed lower Cd uptake than TWC360 and SC128 by recording the lowest Cd content in shoot as well as the lowest BCF and TF (Table 1). Despite the low BCF, TWC324 showed a very high TF.

**Figure 2:**
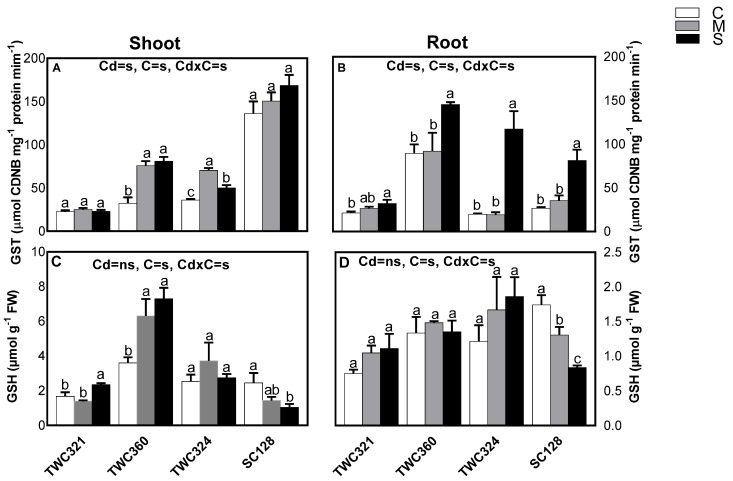
Effect of Cd-stress (C: control, M: mild and S: severe) on (**A**,**B**) activity of glutathione–S-transferase (GST) and (**C**,**D**) glutathione reduced (GSH) of shoot and root of maize cultivars, respectively. Values are expressed as means ± SE (*n* = 3). Bars with at least one similar letter within each cultivar indicate a non-significant difference (*p* ≤ 0.05). Two-way ANOVA was applied to study the effect of the two factors, Cd-stress (Cd) and cultivars (C), as well as their interaction (Cd × C).

To study if the cultivar TWC360 manages Cd detoxification better than sensitive cultivars, we studied the enzyme GST and its substrate GSH to clarify their role in the detoxification of Cd in the investigated cultivars (Figure 2A–D). The activity of GST enzyme was amplified in the root of all the tested cultivars, particularly TWC324. While in shoots, this enzyme was stimulated in TWC360 but changed non-significantly in most of the other cultivars (Figure 2A,B). Compared to plant roots, GSH concentrations were augmented in shoots of the tolerant cultivars and the augmentation was more dramatic in TWC360 than TWC321. In contrast, the moderately sensitive cultivar TWC324 did not significantly change GSH level and the most sensitive one dramatically declined its content of GSH in both organs (Figure 2C,D).

### 3.3. Cd Differentially Induced Oxidative Stress and Antioxidant Defense System in Tolerant and Sensitive Cultivars

**Figure 3:**
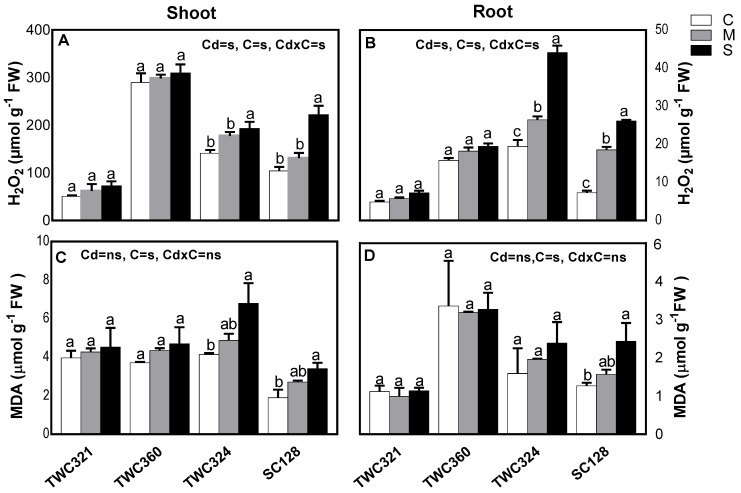
Effect of Cd-stress (C: control, M: mild and S: severe) on (**A**,**B**) hydrogen peroxide (H_2_O_2_) and (**C**,**D**) malondialdehyde (MDA) content of maize shoot and root, respectively. Values are expressed as means ± SE (*n* = 5). Bars with at least one similar letter within each cultivar indicate non-significant difference (*p* ≤ 0.05). Two-way ANOVA was applied to study the effect of the two factors, Cd-stress (Cd) and cultivars (C), as well as their interaction (Cd × C).

Generating free radicals and active oxygen species on exposure to Cd results in oxidative stress that led to lipid peroxidation [57]. In this study, we estimated the level of H_2_O_2_ and lipid peroxidation in the investigated cultivars in response to Cd-induced oxidative stress and membrane damage. H_2_O_2_ content significantly increased in shoots as well as roots of SC128 and TWC324, whereas TWC360 and TWC321 were not affected (Figure 3A,B). Lipid peroxidation, determined by MDA content was not affected in the tolerant TWC360 and TWC321 shoots and roots (Figure 3C,D), likely by exhibiting antioxidant defenses and explaining their biomass stability under the increased Cd levels. In contrast, there was a significant increase in MDA content in the sensitive SC128 shoot and root as well as TWC324 shoot, indicating the susceptibility to Cd-induced oxidative stress. The intensified generation of H_2_O_2_ and the accumulated MDA, indicating high levels of lipid peroxidation and membrane damage in TWC324 and SC128, were consistent with their overall growth response (Figure 1A–D).

**Figure 4:**
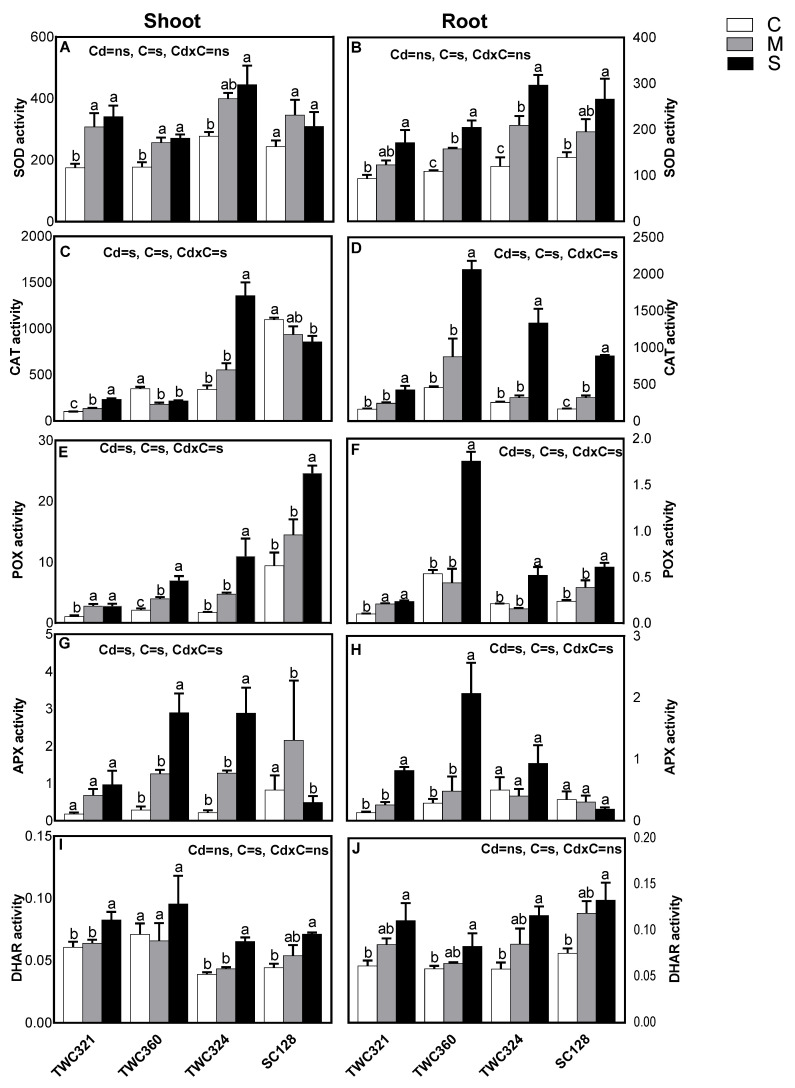
Effect of Cd-stress (C: control, M: mild and S: severe) on activity of (**A**,**B**) super oxide dismutase (SOD;U SOD mg^−1^ protein min^−1^), (**C**,**D**) catalase (CAT; μ mol H_2_O_2_ mg^−1^ protein min^−1^ ), (**E**,**F**) peroxidase (POX; mmol pyrogallol mg^−1^ protein min^−1^), (**G**,**H**) ascorbate peroxidase (APX; μmol ASC mg^−1^ protein min^−1^) and (**I**,**J**) dehydroascorbate reductase (DHAR μmol ASA mg^−1^ protein min^−1^) of shoot and root of maize cultivars, respectively. Values are expressed as means ± SE (*n* = 3). Bars with at least one similar letter within each cultivar indicate non-significant difference (*p* ≤ 0.05). Two-way ANOVA was applied to study the effect of the two factors, Cd-stress (Cd) and cultivars (C), as well as their interaction (Cd × C).

To reveal whether the tolerance of TWC321 and TWC360 under Cd-stress was associated with their modulation of the antioxidant defenses, we measured the activity of SOD, CAT and POX and their efficiency to scavenge the resultant ROS (Figure 4A–F). The activity of SOD varied among the respective organs of the investigated cultivars, particularly shoots, as a response to Cd exposure. In shoots, the activity of SOD was stimulated significantly in tolerant cultivars, TWC321 and TWC360, in response to Cd-stress. The moderately sensitive cultivar TWC324 exhibited a significant stimulation of SOD only at severe stress, while SC128 did not show any significant change (Figure 4A). Additionally, SOD stimulation in tolerant cultivars was observable at both mild and severe stress, indicating that the tolerant cultivars started to induce SOD at the threshold of Cd-stress. The strongest increase was monitored in TWC321 cultivar. Our data also suggested that SOD activity was already stimulated at the lower stress level and in the cultivars with less Cd accumulation. The activity of SOD was increased in roots of all four cultivars, and the increment was more in the sensitive ones (Figure 4B). While TWC360 dramatically increased the activity of CAT and POX in root, it significantly decreased the former and stimulated the later in its shoot (Figure 4C–F). Notably, the cultivar SC128 showed a similar, but weaker response. Both TWC321 and TWC324 stimulated CAT and POX in their organs and the stimulation was stronger in shoots. As two significant enzymes controlling the ascorbate-glutathione cycle with importance in the antioxidant defense, we also investigated DHAR and APX (Figure 4G–J). The cultivar TWC360 strongly increased APX activity in both its shoot and root, stimulated root DHAR and non-significantly changed the same enzyme in the shoot. In contrast, SC128 significantly decreased its APX, while increased DHAR levels in both organs.

### 3.4. Principal Component Analysis (PCA) Confirmed Cultivar Specific Responses

**Figure 5:**
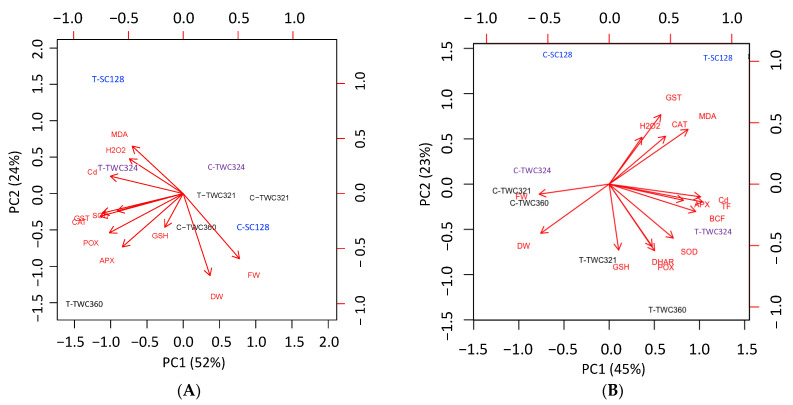
Principal component analysis (PCA) representing the contribution of biochemical parameters of (**A**) shoot and (**B**) root of maize tolerant (TWC321, TWC360) and sensitive (SC324, SC128) cultivars.

For more clarification, we performed two independent principal component analyses for each of the shoot and root (Figure 5A,B) to validate the cultivar specific responses and to reveal whether there is a clear separation between the defense mechanisms across the four contrasting cultivars based on the measured parameters under Cd-stress. For shoots, PCA1 represented 45% of data variances and indicated a separation of the cultivars according to the exposure to Cd-stress (Figure 5A). Regardless of the tolerance of the cultivars, all the non-stressed were gathered on the left half, while their respective Cd-treatments (average of mild and severe stress) were on the right half. The oxidative stress and the defense responses were highly significant to Cd-exposure. The separation with PCA2 (represented 23% of variances) was based on the tolerance of cultivars and the defense mechanisms managed by those cultivars. The treated sensitive cultivar (T-SC128) was located on the upper side with a positive correlation with the oxidative stress parameters (MDA and H_2_O_2_), consistent with our biochemical results. While, the treated tolerant cultivars (T-TWC321 and T-TWC360) and the moderately sensitive one (T-TWC324) were located on the lower side of PCA2, according to their managing detoxification and antioxidant defense (POX and SOD) under Cd stress. Similar to shoot, the separation along PCA1 in case of root (represented 52% of variances) was according to Cd-exposure and separated the responses of control and treated cultivars (Figure 5B). Cd-treated cultivars were positively correlated with the oxidative stress and antioxidant defenses as well as the detoxification parameters. The PCA2 represented 24% of the variation in responses and separated the sensitive cultivars in the upper half from the tolerant ones in the lower half. The sensitive cultivars showed a positive correlation with the oxidative stress parameters (H_2_O_2_ and MDA), while the tolerant cultivars showed a positive correlation with the detoxification mechanisms (GST and GSH) and the antioxidant enzymes, including SOD. Interestingly, in both principal component analyses for shoot and root, the stressed tolerant cultivar (T-TWC321) located beneath its non-stressed respective one (C-TWC321) due to its less sensitivity and Cd content as well as its high maintenance of biomass under Cd-stress. Overall, PCA analysis indicated that SOD and APX (as antioxidant defenses) and the stimulation of GST in addition to the accumulation of GSH (as detoxification mechanisms) are probably the defense strategies that the tolerant cultivars use to cope with Cd-stress.

### 3.5. SOD Overexpression Increased Cd-Stress Tolerance

**Figure 6:**
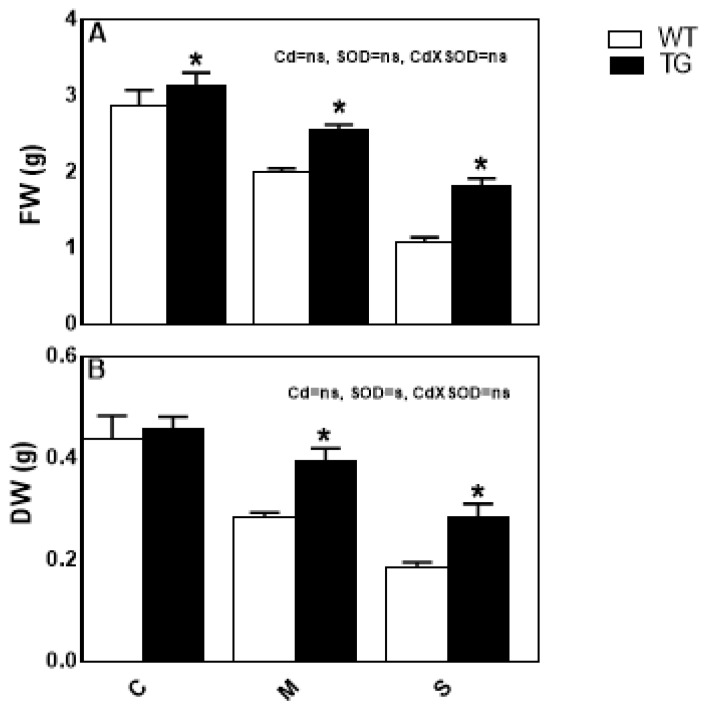
Effect of Cd-stress levels (C; control, M; mild and S; severe) on (**A**) fresh weight (FW) and (**B**) dry weight (DW) of the *FeSOD* overexpressing maize transgenic line (TG) and its wild type (WT). Values expressed as means ± SE (*n* = 5). Bars with (*) indicate significant difference. Two-way ANOVA was applied to study the effect of the two factors, Cd-stress (Cd) and SOD overexpression (SOD), as well as their interaction (Cd × SOD).

**Figure 7:**
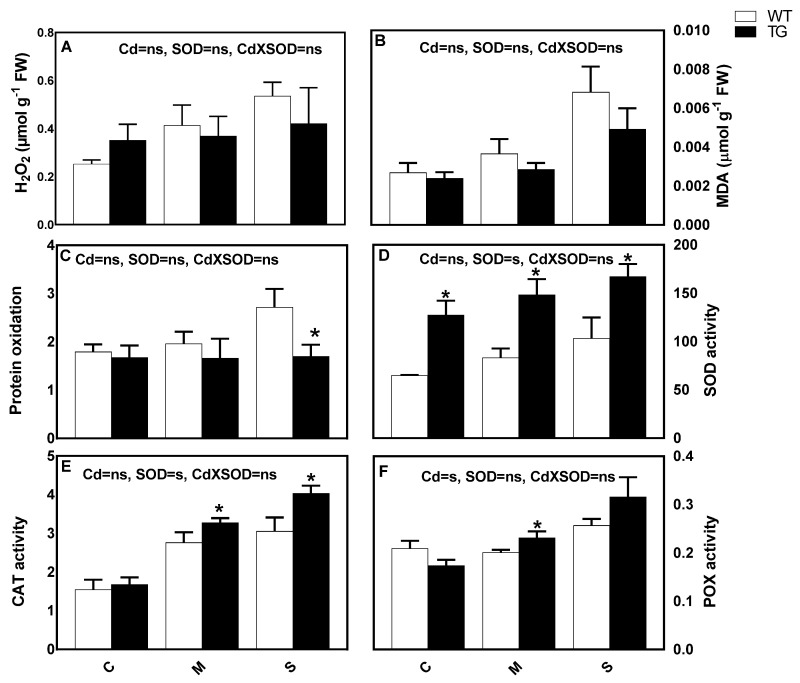
Effect of Cd-stress (C: control, M: mild and S: severe) on (**A**) hydrogen peroxide (H_2_O_2_), (**B**) malondialdehyde (MDA) content, (**C**) protein oxidation, (**D**) superoxide dismutase (SOD;U SOD mg^−1^ protein min^−1^) activity, (**E**) catalase (CAT; μmol H_2_O_2_ mg^−1^ protein min^−1^) activity and (**F**) peroxidase (POX; mmol pyrogallol mg^−1^ protein min^−1^) activity of the *FeSOD* overexpressing maize transgenic line (TG) and its wild type (WT). Values expressed as means ± SE (*n* = 5). Bars with (*) indicate significant difference. Two-way ANOVA was applied to study the effect of the two factors, Cd-stress (Cd) and SOD overexpression (SOD), as well as their interaction (Cd × SOD).

Superoxide dismutase (SOD) is considered the first line of defense responsible for scavenging ROS by dismutating O_2_^•−^ to H_2_O_2_ and O_2_ [58,59]. Our results implicated the role of SOD enzyme in plant resistance to Cd stress. Thus, we conducted an experiment to confirm if the SOD enzyme activity is significantly involved in Cd-stress tolerance. To this end, we grew the overexpressing *FeSOD* maize transgenic line under Cd-stress to investigate its growth, physiology and chemical responses. We compared the performance of shoots of the transgenic line *AtFeSOD* (TG) and the wild type (WT) under Cd-stress. At normal conditions, there was no significant difference between WT and TG in all the investigated parameters, except a higher SOD activity (Figure 6 and Figure 7, Table 2 and Table 3). Under increasing Cd-stress, the TG exhibited a smaller reduction in its FW and DW (Figure 6A,B).

As a result of Cd stress, the stomatal conductance (*gs*) decreased in both WT and TG, but to a lesser extent in TG (Table 2). Under Cd stress, TG showed a smaller decline in total chlorophyll content (Chl a + b), while WT exhibited a progressive increase in the upregulation of carotenoids that were documented as photosynthetic pigments and powerful antioxidants [60]. Inhibition of photosynthesis as a result of Cd exposure was more obvious in WT, which could be linked to its lower total Chl content and photochemical efficiency (Fv/Fm) with respect to TG across the increased Cd-stress (Table 2).

The accumulation of Cd increased by increasing the stress level in both WT and TG (Table 3); nevertheless, TG accumulated less Cd than WT. Interestingly, the transgenic line increased the activity of both GPX and GST, significantly exceeding those of WT in response to severe Cd-stress (Table 3). Moreover, the accumulation of phytochelatins was much higher in TG than in WT at severe Cd-stress (Table 3).

### 3.6. Stimulation of SOD Activity Is Associated with Lower Cd-Induced Oxidative Stress and Stimulation of Antioxidant Responses

Although non-significantly, the concentration of H_2_O_2_ was lower in TG than WT, correlating with a significantly lower accumulation of MDA, revealing a better protection of membranes under Cd-stress in TG. Meanwhile, there was an obvious progression in protein oxidation in WT, while for TG, this parameter did not significantly change by Cd treatment, indicating more protection of the structural and functional proteins of TG and a more efficient ROS—scavenging system. At all Cd concentrations, TG shoots exhibited a higher up regulation of SOD than WT (Figure 7D). Moreover, more enhancement in the activities of CAT and POX was found in TG compared with those of WT under the highest dose of Cd (Figure 7E,F). Most ASC-GSH redox enzymes such as APX, MDHAR, DHAR and GR were stimulated in both WT and TG in response to Cd stress, with a more distinct stimulation in TG (Table 4). The ‘ferric reducing antioxidant power’ (FRAP) content significantly increased in TG under severe Cd-stress; however, there was no observable increase for WT compared to the respective control (Table 4). Flavonoids are powerful antioxidants located in both chloroplast and vacuole and directly scavenge H_2_O_2_, ^•^OH and ^1^O_2_, hence protecting membranes and chloroplast from the oxidative stress [61]. Under severe Cd-stress the increase in TG flavonoids reached 42.25% over respective control, on the other hand there was no notable increase in WT. The components of ascorbate-glutathione cycle were non-significantly changed between shoots of the wild type and the transgenic line (Table 4). Both types, WT and TG, induced a gradual increase in ASC and TASC contents, while they decreased their ASC/TASC ratio due to the increased Cd-stress. However, ASC/TASC ratio was significantly higher in TG relative to WT under severe Cd-stress. The wild type accumulated more TGSH under Cd-stress than the transgenic line. Nevertheless, the GSH/TGSH ratio was higher in TG at the highest concentration of Cd (Table 2), indicating more balance in the redox status and less oxidative stress in TG. Tocopherols, known to scavenge ^1^O_2_, conferring protection to membrane lipids in chloroplast [60], increased as a result of Cd incorporation but differed non-significantly between shoots of TG and WT (Table 2), indicating that the overexpression of *AtFeSOD* did not induce a significant difference in the generation of ^1^O_2_.

## 4. Discussion

Cd is one of the most toxic nonessential heavy metals with no known biological function [62]. Its phytotoxicity threshold varies significantly between plant species and ecotype, and even among different cultivars [63]. For instance, Cd hyper accumulators have shown less Cd toxicity symptoms, such as biomass reduction, chlorosis and necrosis [64,65]. In the current study, 12 Egyptian maize cultivars were grown under Cd-stress to test their Cd stress tolerance. Out of these cultivars, tolerant (TWC360 and TWC321), moderately sensitive (TWC324) and sensitive (SC128) cultivars were selected. In this regard, Cd-induced growth inhibition was less pronounced in TWC360 and TWC321.

The variability in biomass of the four cultivars probably was linked to their potential to uptake and accumulate Cd in the shoot/root. The four cultivars showed differential Cd uptake and accumulation, where cultivars with less Cd accumulation in their shoot, accompanied with stability of biomass, modulated the antioxidant defense mechanisms more effectively to cope with Cd-stress. These results indicate that the tolerance of cultivar TWC360 may be associated with the restricted Cd translocation to the shoot, since tolerant genotypes were reported to have a lower metal content in their shoots and a lower shoot: root Cd ratio than sensitive ones [2]. The higher root biomass of TWC360, regardless of its higher Cd content, indicates that TWC360 in contrast to SC128 efficiently employs mechanisms to detoxify, sequester or compartmentalize Cd accumulated in its roots. Different from TWC360, tolerance of TWC321 could be associated with its lower Cd uptake, in addition to the lowest BCF and TF. All may lead to more stabilized membranes, lower oxidative damage and less inhibited growth compared with TWC324 and SC128. Reduced BCF could be the result of a reduced uptake that might be via precipitating or complexing of Cd in the root environment, or by cellular exclusion [66,67] Although both TWC360 and TWC321 showed a high Cd-tolerance, they differentially induced Cd uptake and accumulation in the shoot, and consequently modulated different tolerance mechanisms. The less-accumulator TWC321 tended to utilize its antioxidant defenses, showing the highest stimulation of SOD in the shoot and increased CAT, POX and DHAR activity in both shoot and root. This cultivar also showed maintenance of the redox status of ascorbate (e.g., stimulating DHAR), showing the most effective ROS scavenging system and resulting in integrated membranes and maintained growth. The effectiveness of antioxidative enzymes SOD, CAT, POX, APX and DHAR in scavenging ROS that result from heavy metal toxicity has been documented [34,68]. On the other hand, the cultivar TWC360 showed more Cd uptake and more accumulation in both shoot and root as compared with TWC321. This higher ability of Cd accumulation was concomitant with induced detoxification and sequestration mechanisms as revealed by high stimulation of GST and accumulation of GSH. In this context, several studies documented the effectiveness of reduced glutathione (GSH) in heavy metal stress tolerance. It efficiently scavenges ^1^O_2_, H_2_O_2_ and ^•^OH [69,70,71], acts as metals ligand in cytosol [72,73] as one of the detoxification reactions and transports to vacuole [74]. In contrast to TWC321 and TWC360, the sensitive cultivar SC128 showed inefficiency in managing either antioxidant defense, SOD, CAT and APX, resulting in its membrane damage, or detoxification by inhibiting GST activity and GSH content. A strong relationship between Cd toxicity increment and the decrease in GSH content has been reported [74]. The severe depletion of GSH content in sensitive cultivars might also be due to its intensive oxidation into GSSG causing unbalance in the redox status of the cell [75]. Furthermore, although the root of SC128 induced stimulation of most of the antioxidant enzymes, including SOD, this response was not enough to rescue its membranes or organelles. This can probably be attributed to the unbalance between H_2_O_2_ production by (SOD) and the activity of H_2_O_2_ degrading enzymes CAT, POX, APX [76]. The root of TWC360 showed efficiency in managing both strategies, antioxidant defense and detoxification mechanisms. The former was revealed by stimulating all the investigated antioxidant enzymes and the later by the stimulated detoxification system (GST). Consequently, compared to sensitive maize cultivars, TWC321 and TWC360 tolerant cultivars showed less oxidative damage. The less sensitivity of TWC324 might come because of its ability to scavenge ROS by the stimulated SOD, CAT and POX. Moreover, as revealed by PCA (Figure 5), the harmful effect of the lower Cd accumulation, compared with SC128, was slightly mitigated by the employment of the antioxidative enzymes SOD, POX and APX showing a less sensitive behavior than SC128.

In addition to its potential role in Cd-stress mitigation according to our results, existence of a maize transgenic line overexpressing an *Arabidopsis* gene for FeSOD [35,77] allowed a direct investigation of the link between antioxidant activity and Cd-stress tolerance. Several studies have already related the enhanced performance of plants to increased antioxidant levels under stress conditions [1,78,79]. For instance, it was reported that maize tolerance to Cd-stress is coupled with SOD activity [29,80]. Similarly, we found that transgenic maize overexpressing *AtFeSOD* (TG) showed enhanced tolerance and improvement of the plant growth and development under Cd stress. That response might be implemented through the activation of antioxidant system resulting in high ROS scavenging and less oxidative damage (lower MDA and protein oxidation) as well as enhanced photosynthesis (higher Chl a + b and Fv/Fm).

The TG also showed a lessening in the accumulation of Cd in its leaves. Consistent with a previous study [81], the enhanced tolerance of *AtFeSOD* overexpressing maize coupled with a declined Cd in shoot suggests that a relation between the balanced generation of ROS and the controlled accumulation of Cd in shoots exists. The enhanced functionality of proteins, resulting from the lower protein oxidation, and the more integrated membranes indicated by lower lipid peroxidation could confer more control on membrane permeability. Lessening Cd accumulation also could be due to the decrease in the transport of Cd to shoot. The enhanced photosynthesis probably supplied assimilates and produced more energy necessary for restricting Cd uptake and translocation. Additionally, lessened accumulation of Cd in leaves could play an indirect role in the increased content of chlorophyll. In this regard, Cd may induce a competitive inhibition of the absorption of some elements that are essential for chlorophyll biosynthesis or by the dysfunction of the enzymes associated with chlorophyll synthesis by interacting with sulfhydryl-rich regions [31,82].

The SOD enzyme is the most vital superoxide anion scavenger that catalyzes converting of O_2_^•−^ to H_2_O_2_. In order to prevent H_2_O_2_ from generating the highly reactive and toxic radical (^•^OH), the plant employs CAT, POD, APX and GPX enzymes to reduce H_2_O_2_ to H_2_O [60]. Thus, the overexpression of *AtFeSOD* also enhanced the plant ability to increase the activity of other antioxidant enzymes. For example, CAT, POD and GPX activities were increased in the TG line over those of WT exposed to Cd stress. That response indicates a vital role of those enzymes in detoxification of ROS [83,84,85]. Moreover, in response to the increased Cd level, both WT and TG increased their detoxification system. In many plant species, the synthesis of PCs is stimulated by Cd exposure and Cd is often sequestered in vacuoles as Cd-phytochelatins complex [86,87,88]. Chelation of heavy metal ions with PCs and sequestering the resultant nontoxic chelates in plant vacuoles are two of the detoxification mechanisms employed by the plant tolerant to heavy metal stress [82]. Apparently the deficiency of PCs production in *pcs* mutant decreased Cd tolerance compared to WT plants [89], while mutant plants overproduced PCs showed a higher tolerance against Cd-stress [89]. Moreover, the higher stimulation of GST shown by TG than WT reveals an activated sequestration of PCs in vacuole as well as the removal of the toxic lipid peroxides [19]. It seems that the thiol-tripeptide (GSH) intensified its versatile roles in the enhanced tolerance of TG. Thus, the tolerance is not only linked to the Cd accumulation, but rather linked to the extent in which the antioxidant and detoxification mechanisms will be sufficient to decrease oxidative stress in Cd-stressed maize plants.

## 5. Conclusions

Cadmium interrupts metabolic homeostasis and causes inactivation of proteins, thereby inducing excessive ROS generation. By studying the response of maize cultivars varying in tolerance to Cd-stress, we conclude that the reduction in biomass of sensitive cultivars was due to the failure of managing either antioxidant or detoxification mechanisms. The tolerant cultivar had lower Cd accumulation and showed a more efficient ROS scavenging system and detoxification mechanisms. Studying the response of an *AtFeSOD overexpressing* transgenic maize line highlighted the role of SOD in Cd-tolerance. The transgenic line showed improved growth and tolerance, less Cd content and enhanced photosynthesis, as well as a stimulated antioxidant defense and detoxification mechanisms. The significant role of SOD in Cd-tolerance was therefore indicated. However, further studies are needed to reveal whether that significance is associated with Cd-accumulation.

## Figures and Tables

**Table 1:** Effect of Cd-stress (C: control, M: mild and S: severe) on shoot and root Cd content, bioconcentration factor (BCF) and translocation factor (TF) of maize cultivars.

Parameter	Cd-Stress	Cultivars			
		TWC321	TWC360	TWC324	SC128
Shoot Cd content (mg kg^−1^)	Control	0.20	3.70	1.20	1.80
	Mild	34.3	59.3	38.0	59.3
	Severe	43.1	72.3	58.1	91.5
Root Cd content (mg kg^−1^)	Control	0.40	2.60	24.7	1.50
	Mild	102.7	121.7	125.6	134.5
	Severe	187.5	240.4	172.9	234.3
Bio-concentration factor	Control	2.14	22.5	92.5	11.85
	Mild	6.21	8.61	7.71	9.81
	Severe	5.71	7.12	5.48	7.44
Translocation factor	Control	0.50	1.42	0.049	1.2
	Mild	0.33	0.49	0.0302	0.441
	Severe	0.23	0.3	0.336	0.391

**Table 2:** Response of photosynthesis of the *FeSOD* overexpressing maize transgenic line (TG) and its wild type (WT) to cadmium stress.

Parameter	Lines	Cadmium Stress			Two-Way ANOVA		
		Control	Mild	Severe	SOD	Cd	SOD × Cd
Photosynthesis	WT	0.14 ± 0.024	0.055 ± 0.011	0.022 ± 0.01	0.383	0	0.993
(mmol CO_2_ m^−2^ s^−1^)	TG	0.152 ± 0.03	0.07 ± 0.02	0.03 ± 0.01			
Chl a + b	WT	0.24 ± 0.05	0.11 ± 0.01	0.06 ± 0.002	0.94	0	0.458
(μg pigment g^−1^ FW)	TG	0.20 ± 0.05	0.12 ± 0.01	0.1 ± 0.01 *			
Carotenoids	WT	0.03 ± 0.004 *	0.04 ± 0.003 *	0.05 ± 0.004	0	0	0.085
(μg pigment g^−1^ FW)	TG	0.014 ± 0.002 *	0.015 ± 0.001 *	0.025 ± 0.002 *			
*gs*	WT	192.46 ± 15.75	63.73 ± 3.96	28.48 ± 2.14	0.772	0	0.006
(mmol m^−2^ s^−1^)	TG	151.32 ± 16.42 *	80.43 ± 7.30 *	45.83 ± 1.9 *			
Fv/Fm	WT	0.81 ± 0.01	0.67 ± 0.026	0.55 ± 0.018	0	0	0.001
	TG	0.82 ± 0.01	0.77 ± 0.01 *	0.70 ± 0.01 *			

Total chlorophyll (Chl a + b), carotenoids, stomatal conductance (*gs*), chlorophyll fluorescence (Fv/Fm). Values expressed as means ± SE (*n* = 8–12). Values with * indicate significant difference. Two-way ANOVA was applied to study the effect of the two factors, Cd-stress (Cd) and SOD overexpression (SOD), as well as their interaction (Cd × SOD).

**Table 3:** Effect of Cd-stress on cadmium content (Cd) and its detoxification in the *FeSOD* overexpressing maize transgenic line (TG) and its wild type (WT).

Parameter	Lines	Cadmium Stress			Two-Way ANOVA		
		Control	Mild	Severe	SOD	Cd	SOD × Cd
Cd	WT	27.77 ± 2.55	40.82 ± 3.50	60.61 ± 2.68	0.14	0	0.13
(mg kg^−1^ FW)	TG	29.57 ± 2.27	38.37 ± 2.51	51.41 ± 2.34 *			
PCs	WT	0.93 ± 0.07	1.30 ± 0.22	1.96 ± 0.14	0.973	0.044	0.558
(μmol g^−1^ FW)	TG	0.73 ± 0.46	1.1 ± 0.1	1.1 ± 0.14 *			
GPX	WT	0.016 ± 0.001	0.018 ± 0.001	0.02 ± 0.003	0.031	0	0.004
(μmol NADPH mg^−1^ min^−1^)	TG	0.015 ± 0.001 *	0.018 ± 0.001	0.04 ± 0.005 *			
GST	WT	0.01 ± 0.001	0.01 ± 0.001	0.01 ± 0.001	0.024	0	0.335
(μmol CDNB mg^−1^ protein min^−1^)	TG	0.01 ± 0.001 *	0.01 ± 0	0.02 ± 0.001 *			

Phytochelatins (PCS), glutathione peroxidase (GPX), glutathione-*S*-transferase (GST). Values expressed as means ± SE (*n* = 6–7). values with * indicate significant difference. Two-way ANOVA was applied to study the effect of the two factors, Cd-stress (Cd) and SOD overexpression (SOD), as well as their interaction (Cd × SOD).

**Table 4:** Effect of Cd-stress on the antioxidant defense of the *FeSOD* overexpressing maize transgenic line (TG) and its wild type (WT).

Parameter	Lines	Cadmium Stress			Two-Way ANOVA		
		Control	Mild	Severe	SOD	Cd	SOD × Cd
APX	WT	0.14 ± 0.004	0.15 ± 0.01	0.17 ± 0.013	0.688	0.012	0.931
(μmol mg^−1^ protein min^−1^)	TG	0.13 ± 0.01	0.15 ± 0.01	0.17 ± 0.01			
MDHAR	WT	0.053 ± 0.003	0.06 ± 0.003	0.06 ± 0.003	0.335	0.02	0.124
(μmol mg^−1^ protein min^−1^)	TG	0.05 ± 0.003	0.06 ± 0.01	0.07 ± 0.004 *			
DHAR	WT	0.01 ± 0.001	0.02 ± 0.001	0.02 ± 0.002	0.912	0	0.049
(μmol mg^−1^ protein min^−1^)	TG	0.01 ± 0.002	0.01 ± 0.001 *	0.027 ± 0.002 *			
GR	WT	0.016 ± 0.001	0.02 ± 0.001	0.03 ± 0.003	0.271	0	0.901
(μmol mg^−1^ protein min^−1^)	TG	0.02 ± 0.001	0.02 ± 0.002	0.03 ± 0.004			
FRAP	WT	14.1 ± 1. 50	14.34 ± 1.25	15.7 ± 0.78	0.007	0.036	0.183
(μmol g^−1^ FW)	TG	17.84 ± 1.78	15.7 ± 0.82	23.68 ± 3.55 *			
Flavonoids	WT	0.62 ± 0.032	0.83 ± 0.04	0.63 ± 0.017	0	0.001	0
(mg quercetin g^−1^ FW)	TG	0.71 ± 0.03	0.78 ± 0.04	1.02 ± 0.03 *			
ASC	WT	1.39 ± 0.20	1.74 ± 0.16	1.80 ± 0.19	0.216	0.083	0.603
(μmol g^−1^ FW)	TG	1.50 ± 0.19	1.82 ± 0.24	2.23 ± 0.18			
DHA	WT	95.73 ± 10.7	115.95 ± 7.60	167.82 ± 8.02	0.63	0.00	0.29
(μmol g^−1^ FW)	TG	102.19 ± 14.77	135.80 ± 9.88	181.5 ± 8.11			
TASC	WT	1.41 ± 0.05	117.69 ± 7.75	183.29 ± 8.20	0.618	0	0.308
(μmol g^−1^ FW)	TG	103 ± 69	137.62 ± 10.05	170.07 ± 8.01			
ASC/TASC	WT	1.41 ± 0.05	1.47 ± 0.047	0.1 ± 0.1	0.321	0.014	0.044
(μmol g^−1^ FW)	TG	1.50 ± 0.04	1.3 ± 0.12	1.3 ± 0.12 *			
GSH	WT	0.18 ± 0.02	0.19 ± 0.018	0.47 ± 0.065	0.063	0	0.292
(μmol g^−1^ FW)	TG	0.27 ± 0.04 *	0.29 ± 0.02 *	0.46 ± 0.04			
GSSG	WT	0.064 ± 0.008	0.12 ± 0.003	0.20 ± 0.04	0.372	0.059	0.145
(μmol g^−1^ FW)	TG	0.08 ± 0.01	0.12 ± 0.02	0.11 ± 0.05			
TGSH	WT	0.24 ± 0.02	0.31 ± 0.02	0.67 ± 0.09	0.339	0	0.041
(μmol g^−1^ FW)	TG	0.36 ± 0.03 *	0.41 ± 0.01 *	0.56 ± 0.11			
GSH/TGSH	WT	73.66 ± 2.5	60.43 ± 2.1	70.04 ± 0.04	0.339	0.12	0.593
(μmol g^−1^ FW)	TG	75.01 ± 4.8	70.17 ± 5.7	81.21 ± 3.85			
Tocopherols	WT	2.71 ± 0.1	3.41 ± 0.11	4.37 ± 0.30	0.701	0.476	0.004
(mg g^−1^ FW)	TG	4.23 ± 0.5 *	3.38 ± 0.40	3.19 ± 0.15 *			

Ascorbate peroxidase (APX), monodehydroascorbate reductase (MDHAR), dehydroascorbate reductase(DHAR), glutathione reductase (GR), FRAP, flavonoids, ascorbate (ASC), dehydroascorbate (DHA), total ascorbate (TASC), ascorbate redox status (ASC/TASC), reduced glutathione (GSH), oxidized glutathione (GSSG), total glutathione (TGSH), glutathione redox status (GSH/TGSH) and tocopherols. Values expressed as averages ± SE (*n* = 3–5) and * indicates a significant difference (*p* ≤ 0.05). Two-way ANOVA was applied to study the effect of the two factors, Cd-stress (Cd) and SOD overexpression (SOD), as well as their interaction (Cd × SOD).

## Data Availability

Data of this study are included in the article and Supplementary Material.

## Supplementary Materials

The following are available online at https://www.mdpi.com/article/10.3390/antiox10111812/s1, Table S1: Tolerance index (TI) in terms of maize shoot and root lengths, fresh weights and dry weights under mild and severe cadmium stress, Figure S1: Effects of cadmium stress (C: control, M: mild, S: sever) on shoot and root lengths (**A**,**B**) of twelve maize cultivars. Values are expressed as averages ± SE (*n* = 7–10). Bars with at least one similar letter within each cultivar indicate non-significant difference (*p* ≤ 0.05), Figure S2: Effects of cadmium stress (C: control, M: mild, S: sever) on shoot and root fresh weight (FW) (**A**,**B**) of twelve maize cultivars. Values are expressed as averages ± SE (*n* = 7–10) ± SE. Bars with at least one similar letter within each cultivar indicate non-significant difference (*p* ≤ 0.05), Figure S3: Effects of cadmium stress (C: control, M: mild, S: severe) on shoot and root dry weight (DW) (**A**,**B**) of twelve maize cultivars. Values are expressed as averages ± SE (*n* = 7–10) ± SE. Bars with at least one similar letter within each cultivar indicate non-significant difference (*p* ≤ 0.05).
