# Supplementary material for: Differences in Cadmium Accumulation, Detoxification and Antioxidant Defenses between Contrasting Maize Cultivars Implicate a Role of Superoxide Dismutase in Cd Tolerance
Source: Antioxidants (Basel). 2021 Nov 15;10(11):1812. doi: 10.3390/antiox10111812 (PMC8614887; doi:10.3390/antiox10111812)
Supplement: Supplementary file 1 [file antioxidants-10-01812-s001.zip › antioxidants-1435686-supplementary.pdf]

Differences in Cadmium Accumulation, Detoxification and Antioxidant Defenses between Contrasting Maize Cultivars Implicate a Role of Superoxide Dismutase in Cd Tolerance

Twelve maize cultivars were tested in pot experiment to select those are the most tolerant and the most sensitive cultivars to Cd-stress. Cadmium was applied, before sowing seeds, as different concentrations (0.0, 80 and 160 mg of $CdCl_2 \cdot 2.5H_2O$ Kg^{-1} soil), representing control, mild and severe Cd-stress, respectively. Seedlings were grown for 50 days. Maize growth was estimated as length and fresh and dry weights of shoot and root. Tolerance index (TI) was estimated for each parameter as following:

TI= [(XWM+XWS)/XWC]/2

Where XWM; parameter estimated at mild stress, XWS; parameter estimated at severe stress and XWC; parameter estimated at control. Cultivars generally scored the highest and lowest shoot and root TI are the most tolerant and sensitive cultivars.

The results indicated that the highest tolerance index was for TWC360 followed by TWC321. Those cultivars exhibited the lowest TI of most of the morphological parameters (Table S1). In contrast, SC128 showed a sensitive behavior and scored the lowest TI of all the tested parameters, in addition to a great inhibition in its shoot (-48.9%) and root (-52.2%) dry biomass (Figure S2) respective to control. The performance of TWC324 was also sensitive but less sensitive than SC128.

Table S1: Tolerance index (TI) in terms of maize shoot and root lengths, fresh weights and dry weights under mild and severe cadmium stress.

Parameter	Cd concentration	**SC128**	SC131	P3444	SC368	**TWC 324**	SC10	SC166	SC176	**TWC360**	**TWC321**	P3433	TWC654
Shoot length	Mild	**0.81**	0.80	0.72	0.91	**0.67**	1.14	0.85	0.94	**0.96**	**1**	1	0.98
	Severe	**0.71**	0.86	0.73	0.79	**0.85**	0.9	0.86	0.81	**0.94**	**1.05**	0.90	0.99
	Average	**0.76**	0.83	0.72	0.85	**0.76**	1.03	0.85	0.88	**0.95**	**1.03**	0.94	1
Root length	Mild	**0.94**	0.51	1.06	1.1	**1.3**	1.12	1.11	1.1	**1.53**	**1.15**	0.97	1.3
	Severe	**0.74**	0.76	0.72	0.76	**1.13**	0.90	1.23	1	**1.68**	**1.30**	0.91	1.2
	Average	**0.84**	0.64	0.89	0.91	**1.21**	1.01	1.17	1.01	**1.60**	**1.22**	0.94	1.2
Shoot FW	Mild	**0.57**	0.77	0.86	0.72	**0.64**	1.15	0.75	0.87	**1.1**	**0.97**	1.1	1
	Severe	**0.47**	0.92	0.73	0.71	**0.73**	0.74	0.80	0.72	**1.03**	**1.24**	0.9	1.03
	Average	**0.52**	0.84	0.80	0.71	**0.69**	0.95	0.77	0.80	**1.1**	**1.10**	1	1
Root FW	Mild	**0.78**	0.69	1.11	0.81	**0.71**	0.97	0.82	0.90	**0.94**	**0.80**	0.90	1.2
	Severe	**0.51**	0.95	0.61	0.83	**0.77**	0.77	1.12	0.90	**1.1**	**1.08**	0.80	0.8
	Average	**0.64**	0.82	0.86	0.82	**0.74**	0.87	0.97	0.90	**1.02**	**0.94**	0.84	1
Shoot DW	Mild	**0.62**	0.63	0.99	0.84	**0.71**	0.79	0.91	0.73	**1.17**	**0.75**	0.93	0.66
	Severe	**0.55**	0.98	0.48	0.74	**0.52**	0.74	0.72	1.04	**1.26**	**0.92**	1.16	0.41
	Average	**0.59**	0.8	0.74	0.79	**0.61**	0.76	0.81	0.88	**1.21**	**0.84**	1.04	0.54
Root DW	Mild	**0.65**	0.69	0.97	0.96	**0.89**	0.87	1.21	0.79	**1.74**	**0.83**	0.75	0.64
	Severe	**0.49**	0.79	0.68	0.63	**0.43**	0.86	1.05	0.79	**1.32**	**1.05**	0.97	0.83
	Average	**0.57**	0.74	0.83	0.8	**0.66**	0.86	1.13	0.79	**1.53**	**0.94**	0.86	0.73

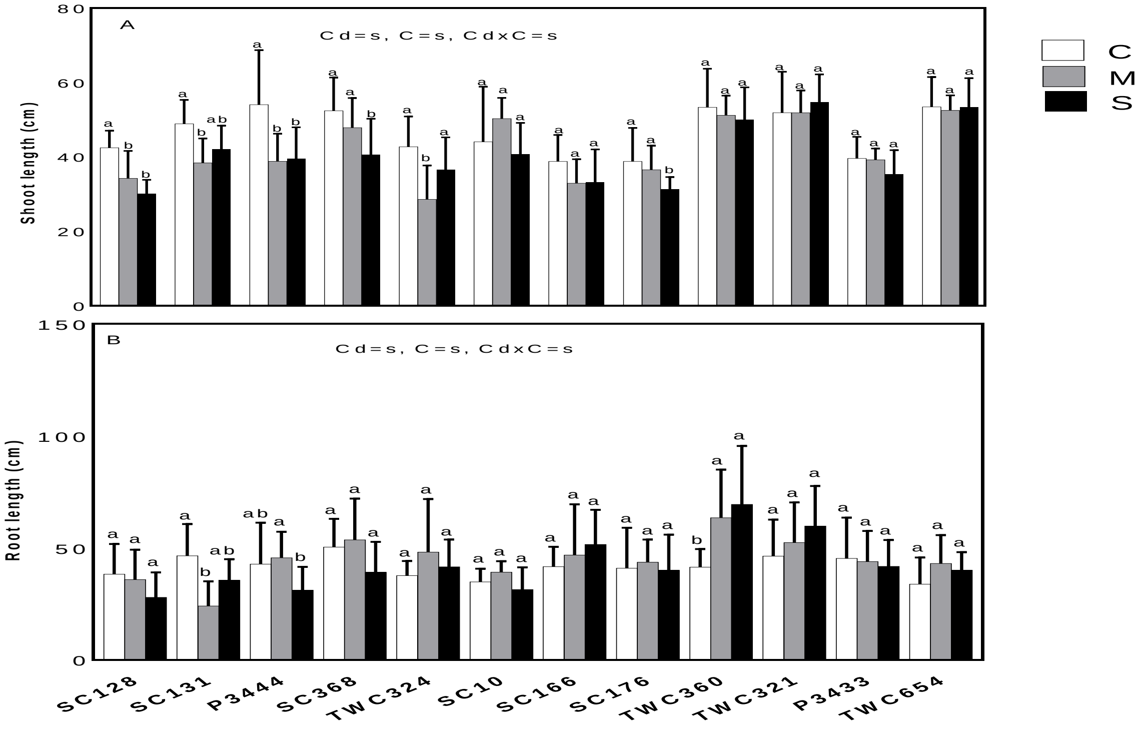

Figure S1: Effects of cadmium stress (C: control, M: mild, S: sever) on shoot and root lengths (**A**,**B**) of twelve maize cultivars. Values are expressed as averages ± SE (n = 7–10). Bars with at least one similar letter within each cultivar indicate non-significant difference ($p \leq 0.05$).

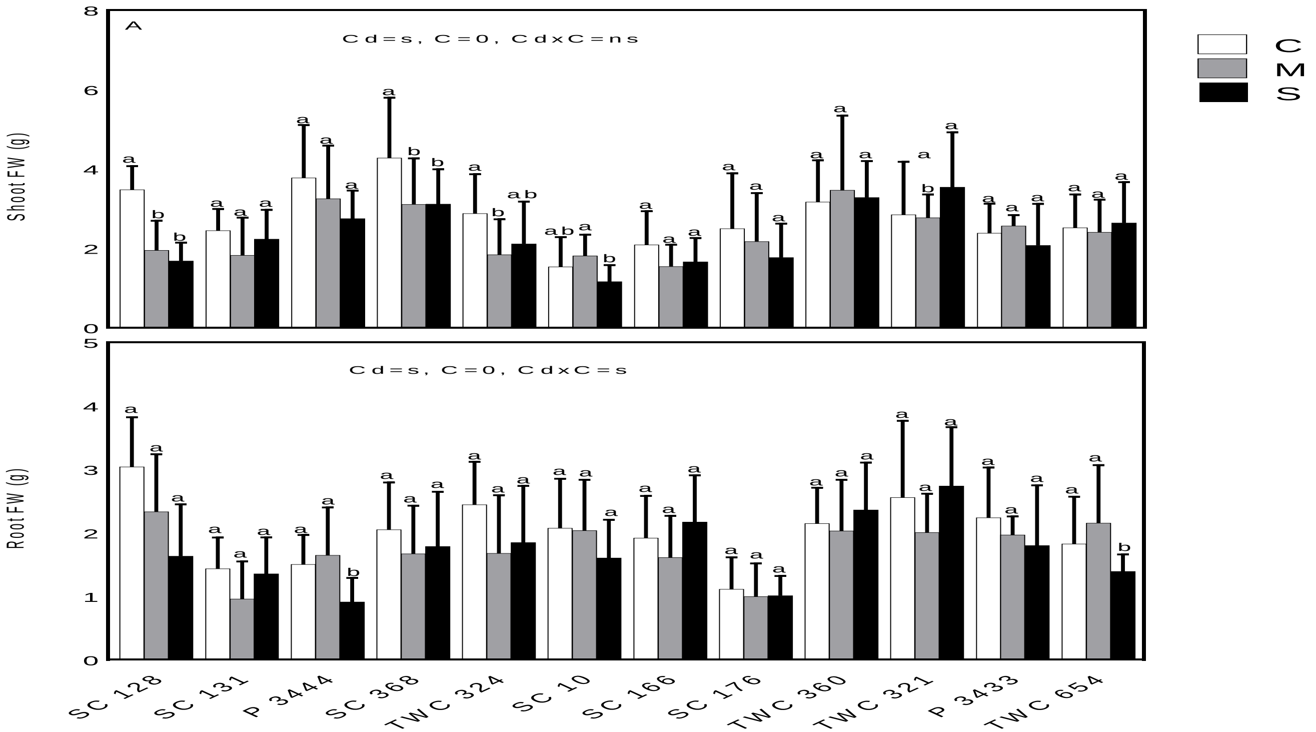

Figure S2: Effects of cadmium stress (C: control, M: mild, S: sever) on shoot and root fresh weight (FW) (**A**,**B**) of twelve maize cultivars. Values are expressed as averages ± SE (*n* = 7–10) ± SE. Bars with at least one similar letter within each cultivar indicate non-significant difference ($p \leq 0.05$).

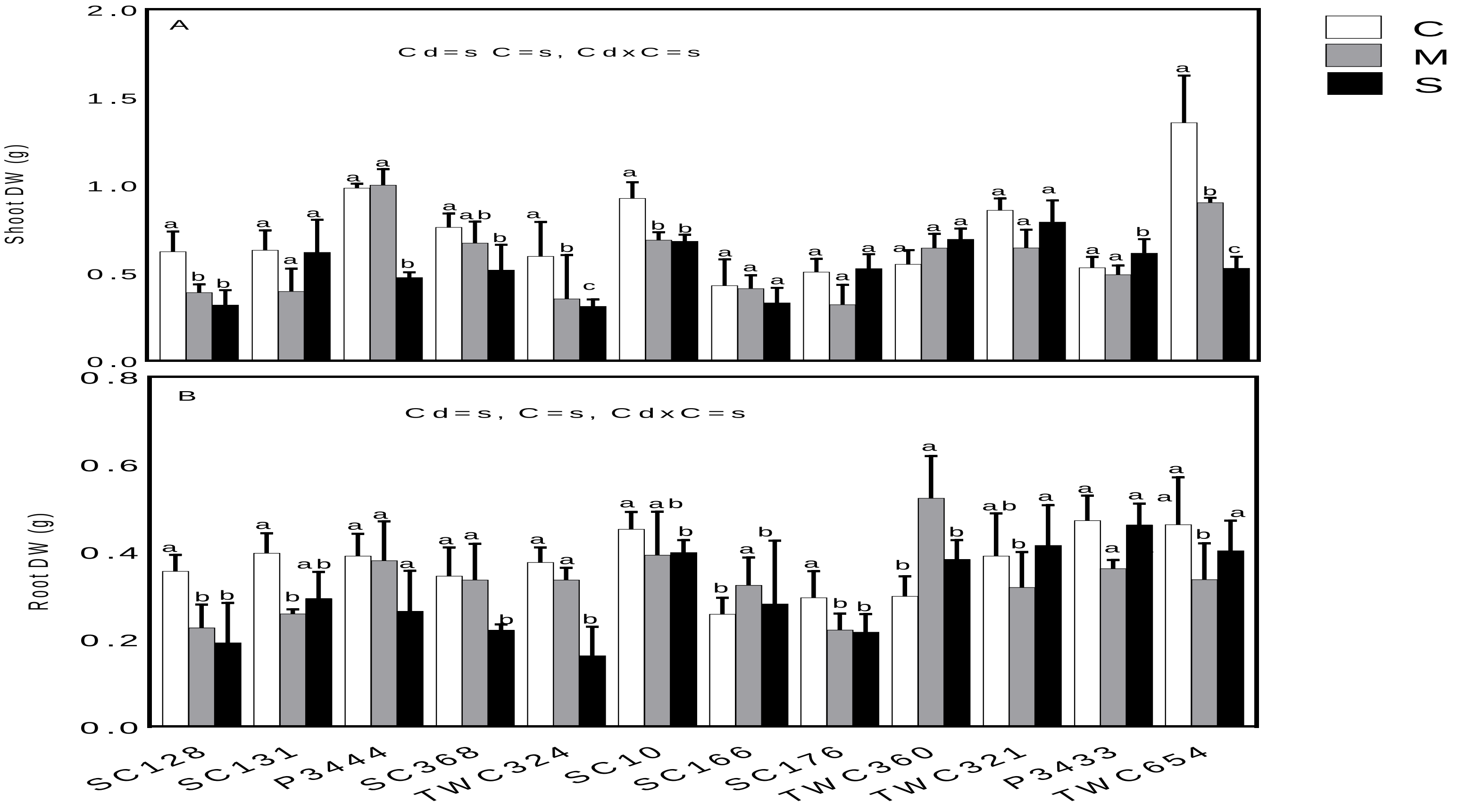

Figure S3: Effects of cadmium stress (C: control, M: mild, S: severe) on shoot and root dry weight (DW) (**A**,**B**) of fifteen maize cultivars. Values are expressed as averages ± SE (n = 7–10) ± SE. Bars with at least one similar letter within each cultivar indicate non-significant difference ($p \leq 0.05$).
